# Differential effects of ketoconazole, fluconazole, and itraconazole on the pharmacokinetics of pyrotinib *in vitro* and *in vivo*

**DOI:** 10.3389/fphar.2022.962731

**Published:** 2022-09-07

**Authors:** Li Wang, Fan Wu, Jia Xu, Yu Wang, Weidong Fei, Hui Jiang, Peiwu Geng, Quan Zhou, Shuanghu Wang, Yongquan Zheng, Huadong Deng

**Affiliations:** ^1^ Department of Pharmacy, Women’s Hospital, Zhejiang University School of Medicine, Hangzhou, China; ^2^ Department of Pharmacy, The First Affiliated Hospital of Zhejiang Chinese Medical University, Hangzhou, China; ^3^ The Laboratory of Clinical Pharmacy, The Sixth Affiliated Hospital of Wenzhou Medical University, Lishui People’s Hospital, Lishui, China; ^4^ Department of Ultrasonography, The Sixth Affiliated Hospital of Wenzhou Medical University, Lishui People’s Hospital, Lishui, China

**Keywords:** pyrotinib, drug-drug interaction, metabolism, pharmacokinetics, CYP3A4, azole antifungal

## Abstract

It has been reported that drug-drug interactions (DDIs) can affect the pharmacokinetics and pharmacodynamics of various oral drugs. To better understand the effects of azole antifungal drugs (ketoconazole, fluconazole, and itraconazole) on pyrotinib’s pharmacokinetics, DDIs between pyrotinib and three azoles were studied with Sprague-Dawley (SD) rat liver microsomes *in vitro*. Additionally, *in vivo* pyrotinib metabolic experiment was also performed. Twenty-four male SD rats were randomly divided into four groups: the ketoconazole (40 mg/kg), fluconazole (40 mg/kg), itraconazole (40 mg/kg), and the control group. UPLC-MS/MS was used for the determination of Pyrotinib’s plasma concentration in rats. *In vitro* experiments showed that IC_50_ values of ketoconazole, fluconazole and itraconazole were 0.06, 11.55, and 0.27 μM, respectively, indicating that these drugs might reduce the clearance rate of pyrotinib at different degrees. In rat studies, coadministration of pyrotinib with ketoconazole or fluconazole could dramatically increase the C_max_ and AUC_(0-t)_ values and decrease the clearance rate of pyrotinib, especially for ketoconazole. However, coadministration with itraconazole had no impact on the pharmacokinetic characters of pyrotinib. These data indicated that ketoconazole and fluconazole could significantly decrease the metabolism of pyrotinib both *in vitro* and *in vivo*. More attentions should be paid when pyrotinib is combined with azole antifungal drugs in clinic although further investigation is still required in future.

## 1 Introduction

Pyrotinib is a potent epidermal growth factor receptor/human epidermal growth factor receptor 2 (EGFR/HER2) inhibitor and can be used to treat HER2-positive breast cancer (Li et al., 2017; Blair, 2018; Huang et al., 2020). Pyrotinib gained its first global conditional authorization in August 2018 in China because of the promising outcome of a phase II trial for use with capecitabine in patients who had previously received anthracycline or taxane chemotherapy for HER2-positive or metastatic breast cancer (MBC) (Blair, 2018). It has been reported that combination of pyrotinib with palbociclib, a CDK4/6 inhibitor, could inhibit the reproductive capacity of several HER2-positive BC human cell lines (Zhang et al., 2019; Iancu et al., 2022). In one human study, pyrotinib was well-tolerated and showed promising efficacy in HER2-positive patients with metastatic breast cancer (Ma et al., 2017).

Pyrotinib’s pharmacokinetic (PK) characters have been previously investigated in many studies (Zhu et al., 2016; Meng et al., 2019). It was reported that the time required to attain maximum plasma concentration (T_max_) following the initial dose of 80–400 mg pyrotinib was 3–5 h, and the mean terminal half-life (t_1/2_) was 11.4–15.9 h. The maximum concentration (C_max)_ and area under the concentration-time curve in steady state (AUCss) of pyrotinib were shown to have a linear PK profile (Ma et al., 2017). It has been reported that pyrotinib is largely metabolized by cytochrome P450 3A4 (CYP3A4) and is primarily eliminated in the feces. SHR150980 (O-depicolyl pyrotinib, M1), SHR151468 (O-depicolyl and pyrrolidine lactam pyrotinib, M2), and SHR151136 (pyrrolidine lactam pyrotinib, M5) were the primary metabolites for pyrotinib and M1 was the major metabolite in human body (Wen et al., 2021). CYP3A4 belongs to the CYP isoform family, one major monooxygenase superfamily, that is, responsible for the drug metabolism. CYP3A4 mediates the metabolism of 45%–60% of currently prescribed medicines, and many inhibitors and activators can alter the drug metabolic activity of CYP3A4 toward specific substrates (Lv et al., 2018). Therefore, the coadministration of pyrotinib with drugs that can modulate CYP3A4 enzyme activity may alter pyrotinib’s exposure time in human body.

Azoles are substrates of cytochrome P450 (CYP) isoenzyme and are also regarded as the typical inhibitors for CYPs that play an important role in various drug-drug interactions (DDIs) (Bellmann and Smuszkiewicz, 2017). Previous PK studies also suggested that phase 1 and 2 biotransformation enzymes, including transporter proteins, play an important role in azole-related drug interactions (Nivoix et al., 2009). Ketoconazole, fluconazole, and itraconazole are the most commonly used azole medications to treat fungal infections in cancer patients (Parasrampuria et al., 2016). These drugs are also reported to be the powerful CYP3A4 and P-gp inhibitors that could inhibit several substrates of other tyrosine kinase inhibitors, including apatinib and sunitinib (Lou et al., 2019; Wang et al., 2021). It was reported that itraconazole could increase pyrotinib’s plasma concentration in healthy Chinese individuals (Liu et al., 2021). However, no data have been reported on the effects of ketoconazole and fluconazole on pyrotinib’s PK parameters, and additional experimental studies should be performed to evaluate the potential DDIs between azole drugs and pyrotinib. The aim of this study was to investigate the interactions between the three azoles and pyrotinib both *in vitro* and *in vivo*. We forecast that the results may be helpful for the clinical safety evaluation of interactions between pyrotinib and three azole antifungal drugs.

## 2 Materials and methods

### 2.1 Chemicals and reagents

Ketoconazole, fluconazole, and itraconazole were obtained from Melone Biotechnology Co. Ltd. (Beijing, China). Pyrotinib and its metabolite, pyrotinib M1 (purity >98%), were obtained from Guangzhou Zero One Biological Technology Co., Ltd. (Guangzhou, China). Midazolam [used as an internal standard (IS)] was obtained from Jiangsu Nhwa Pharmaceutical Co., Ltd. (Jiangsu, China). Formic acid (gradient grade for liquid chromatography) was obtained from Sigma-Aldrich. Methanol (MeOH) and acetonitrile (ACN) (gradient grade for liquid chromatography) were obtained from Merck (Billerica, MA, United States). Purified water was produced by the Milli-Q Plus filtration system (Millipore, Billerica, MA, United States). The coenzyme NADPH was obtained from Roche Diagnostics GmbH (Mannheim, Germany).

### 2.2 Preparation of rat liver microsomes

The rats were anesthetized with 10% chloral hydrate after 12 h of starvation. The rats’ abdominal cavity was opened to expose the liver after disinfection, and 0.15 mol/L KCl buffer (pH = 7.4) was injected through the superior vena cava until the blood was flushed clean. The rat liver tissue was then accurately removed and transferred to Petri dishes at 0–4°C. The tissues were homogenized in 0.15 mol/L KCl-PBS buffer and stored at 4°C. The homogenate (CR30NX, Eppendorf, Germany) was centrifuged at 9000 rpm at 4°C for 30 min. The supernatant was transferred to another tube and was centrifuged at 105000 rpm at 4°C for 60 min. The precipitate containing liver microsomes was removed and suspended in 0.15 mol/L KCl-PBS buffer (containing 0.25 mol/L sucrose) and stored at −80°C for later use. The liver microsomal concentration was 8.838 mg/ml after the determination with the BCA protein quantification kit (Thermo Scientific, Waltham, MA, United States).

### 2.3 Instruments and UPLC-MS/MS conditions

Waters Acquity UPLC I-Class (Waters, USA) was used to separate pyrotinib and midazolam. A binary solvent manager (BSM) and sample manager with a flow-through needle (SM-FTN) were used in the UPLC system. The metabolites’ effective separation was achieved using Acquity UPLC HSS T3 (100 × 2.1 mm, 1.8 μm particle size) (Waters Corporation, Milford, MA, USA) with a mobile phase of acetonitrile (solvent A) and 0.1% formic acid water containing 20 mmol ammonium acetate (solvent B). The gradient program was set as following: 0–0.3 min 10–30% A; 0.3–1 min 30–95% A; 1–2 min 95% A; 2–2.3 min 95–10% A. The mobile phase’s flow rate was 0.4 mL/min, the column temperature was set as 40 °C, and the injection volume was 2 μL. The XEVO TQD triple quadrupole mass spectrometer was equipped with an electrospray ionization (ESI) source, and the multiple reaction monitoring (MRM) mode was selected for quantification. The data were collected using the Mass lynx 4.1 software (Waters Corp.). Mass spectral data were obtained in positive electrospray mode (ESI+) in MRM mode. Nitrogen was used as desolvation (1000 L/h) and cone gases (50 L/h). The ion monitoring voltage conditions were as follows: capillary voltage, 2.5 kV; source temperature, 150°C; desolvation temperature, 500°C. Various reaction monitoring methods were used for quantitative analysis. The pyrotinib, pyrotinib M1, and midazolam transitions are shown in Table 1.

**TABLE 1:** The transition parameters of pyrotinib, pyrotinib M1, and midazolam.

Compound name	Parent (m/z)	Daughter (m/z)	Dwell (s)	Cone (V)	Collision (V)
Pyrotinib	583.16	138.10	0.08	55	30
Pyrotinib M1	492.15	138.10	0.08	55	30
Midazolam	325.98	291.07	0.08	50	26

### 2.4 *In vitro* drug-drug interaction studies in RLMs

200 µl of the incubation mixture was set up as following: 0.3 mg/ml RLMs, 100 mM potassium phosphate buffer (pH 7.4), 1 mM NADPH, and three azole antifungal drugs. A series concentrations of ketoconazole (0.01, 0.05, 0.1, 0.5, 1, 5, and 10 µM) were used to determine its IC_50_ (half maximum inhibitory concentration). For fluconazole and itraconazole, the concentrations of them were all adjusted to 0.01, 0.1, 1, 5, 10, 50, and 100 µM. For the inhibition type determination experiment, a range of pyrotinib (1, 2, 4, and 8 µM) and a series concentrations of ketoconazole (0, 0.03, 0.06, and 0.12 µM), fluconazole (0, 6, 12, and 24 µM), itraconazole (0, 0.15, 0.3, and 0.6 µM) were used for the Lineweaver-Burk Plot analysis and inhibition constants (Ki and αKi) calculation. The ingredients were combined and pre-warmed for 5 min at 37°C. The reaction was initiated with NADPH and incubated at 37°C for 30 min. Then, 200 µl of acetonitrile-midazolam solution (containing midazolam 200 ng/ml) was added and the mixture was vortexed for 2 min to stop the reaction. After been centrifuged at 13000 rpm for 5 min, 2 µl aliquot of the supernatant was then used for the UPLC-MS/MS analysis.

### 2.5 Method validation

The test method was extensively verified regarding precision, accuracy, and stability, according to the EMA (European Medicines Agency) 2011 and FDA (Food and Drug Administration) 2018 criteria. In six replicates, quality control (QC) samples were generated at low, medium, and high concentrations and were utilized to validate the procedure.

### 2.6 Animal experiments

Male Sprague-Dawley rats (180–220 g) were supplied by the Experimental Animal Center of Wenzhou Medical University. The animals were placed in a controlled environment of 20–26°C and 55 ± 15% relative humidity under a 12 h light-dark cycle. Except for the 12-h fasting period before the PK study, all rats were fed a standard rodent diet and freely consumed tap water. All experimental procedures and protocols were reviewed and approved by the Animal Care and Use Committee of the Wenzhou Medical University (No. wydw 2019-650), following the Guidelines for The Care and Use of Laboratory Animals.

### 2.7 *In vivo* pharmacokinetic experiments

**FIGURE 1:**
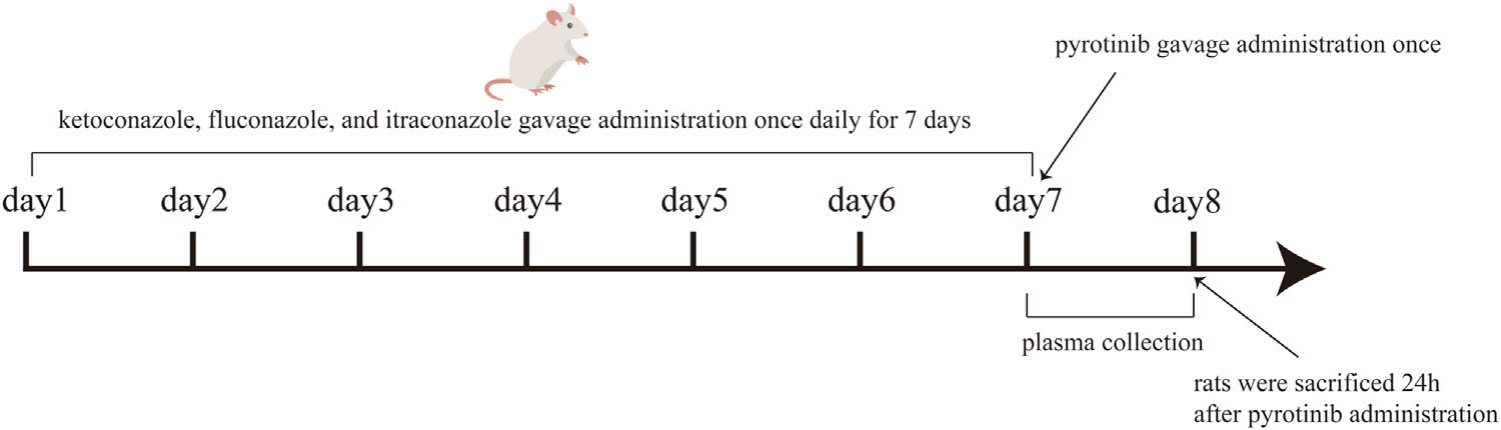
The experimental timeline.

The rats were randomly divided into groups A (control group), B (the multiple dose of 40 mg/kg ketoconazole for consecutive 7 days), C (the multiple dose of 40 mg/kg fluconazole for consecutive 7 days), and D (the multiple dose of 40 mg/kg itraconazole for consecutive 7 days). After the last administration of the three azoles or 0.5% CMC-Na (control group), a single dose of 8 mg/kg pyrotinib was administered orally to all rats in each group. Each group consisted of 6 rats. The azole antifungal medication was dissolved in 0.5% carboxymethylcellulose sodium solution (CMC-Na). The experimental timeline showed in Figure 1. All groups were administered pyrotinib in 0.5% CMC-Na, and blood samples were obtained from the caudal vein and deposited in Eppendorf tubes containing heparin sodium at different times (0.083, 0.25, 0.5, 1, 2, 3, 4, 6, 12, and 24 h). The plasma was obtained by centrifuging the blood samples at 4000 rpm for 10 min at 4°C and stored at −80°C until analysis.

### 2.8 Preparation of plasma samples

The frozen plasma samples were thawed at room temperature and uniformly mixed; 50 μL of plasma sample was removed and mixed with 150 μL acetonitrile-midazolam solution (containing midazolam 200 ng/ml) to precipitate the proteins, followed by vortexing for 30 s and centrifuging at 13,000 rpm for 5 min. Then 150 μL of the supernatant was collected and placed into UPLC vials with 2 μL of sample was subjected to UPLC-MS/MS analysis.

### 2.9 Data analyses

GraphPad Prism 7.0 software (GraphPad Software Inc.) was used to calculate the enzyme kinetic parameters of substrates which include V_max_, K_m_, IC_50_, Ki, and αKi values. The enzyme inhibition mode was obtained using the Linear Lineweaver-Burk plots. The PK characteristics, maximal plasma concentration (C_max_), time to peak plasma concentration (T_max_), apparent volume of distribution (Vz/F), area under the plasma concentration-time curve (AUC), elimination half-life (t_1/2_), plasma clearance (Cl/F), and mean residence time (MRT), were calculated using DAS (Drug and Statistics) software (Version 3.2.8, The People’s Hospital of Lishui, China). The one-way ANOVA and a Dunnet’s multiple range test were used for the statistical comparisons within groups with SPSS software (Version 25.0; SPSS Inc.). Statistical significance was set at *p* < 0.05.

## 3 Results

### 3.1 Method validation

This method was designed to conform to EMA and FDA regulatory requirements for bioanalytical methods. The details of the method validation results were presented in Tables 2, 3.

**TABLE 2:** Evaluation of the intra- and inter-day precision by the proposed UPLC-MS/MS method for determination of pyrotinib and pyrotinib M1 in rat plasma (*n* = 6).

Analytes	Preparation Concentration (μM)	Inter-day		Intra-day	
		Precision RSD (%)	Accuracy RE (%)	Precision RSD (%)	Accuracy RE (%)
Pyrotinib	15	8.34	105.51	8.42	107.87
	150	5.99	107.78	2.27	108.51
	4500	2.55	103.17	1.94	103.02
Pyrotinib M1	0.015	11.64	106.67	9.90	104.07
	0.15	7.81	108.67	5.78	106.07
	0.75	5.00	106.93	3.22	105.77

**TABLE 3:** Stability evaluation results of pyrotinib and pyrotinib M1 in rat plasma under different conditions (*n* = 6).

Analytes	Preparation Concentration (μM) h	Room temperature, 6 h		4°C, 24 h		−80°C, 7 days	
		RSD (%)	RE (%)	RSD (%)	RE (%)	RSD (%)	RE (%)
Pyrotinib	15	10.06	105.41	11.85	102.40	9.11	98.95
	150	3.47	106.18	9.66	107.05	7.58	108.01
	4500	2.63	103.56	5.91	101.04	4.69	100.79
Pyrotinib M1	0.015	9.82	102.22	7.73	104.44	10.20	109.33
	0.15	5.79	102.44	4.23	108.67	4.36	107.56
	0.75	7.61	108.42	1.94	103.58	2.87	104.47

### 3.2 Effects of ketoconazole, fluconazole, and itraconazole on the metabolism of pyrotinib *in vitro*

**FIGURE 2:**
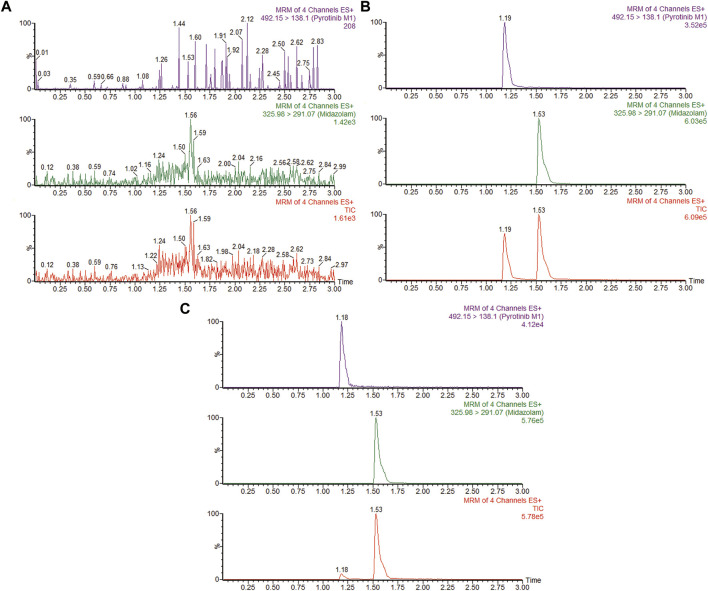
MRM chromatograms of pyrotinib M1 and IS in RLMs. **(A)** Blank RLMs **(B)** Denatured RLMs with pyrotinib M1 and IS, and **(C)** Incubation sample.

**FIGURE 3:**
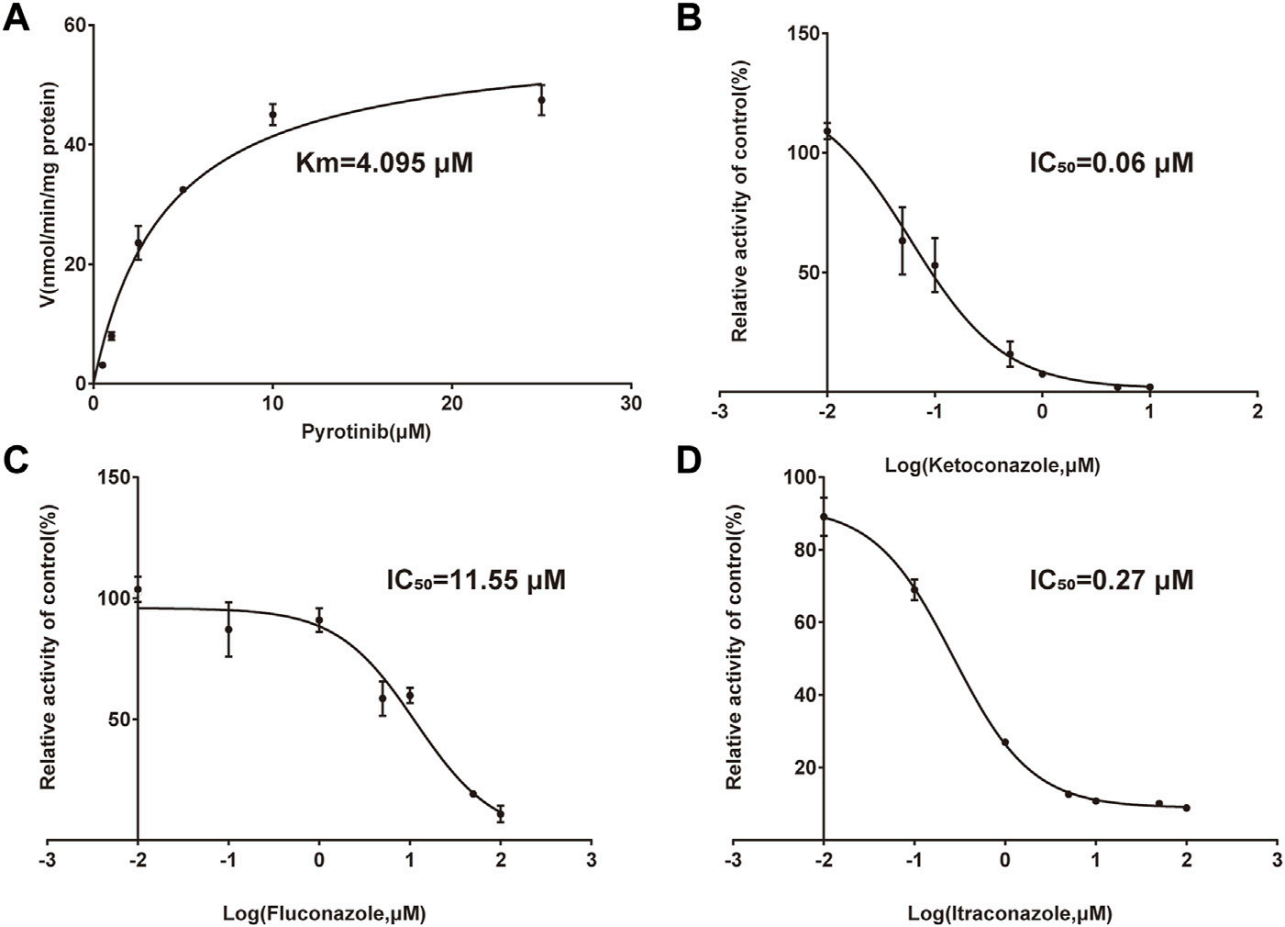
Michaelis-Menten curve of pyrotinib (0.5–25 μM) metabolism in RLMs **(A)**. The half-maximal inhibitory concentration (IC_50_) curve of ketoconazole **(B)**, fluconazole **(C)**, and itraconazole **(D)** (values are means ± standard deviations, *n* = 3).

**FIGURE 4:**
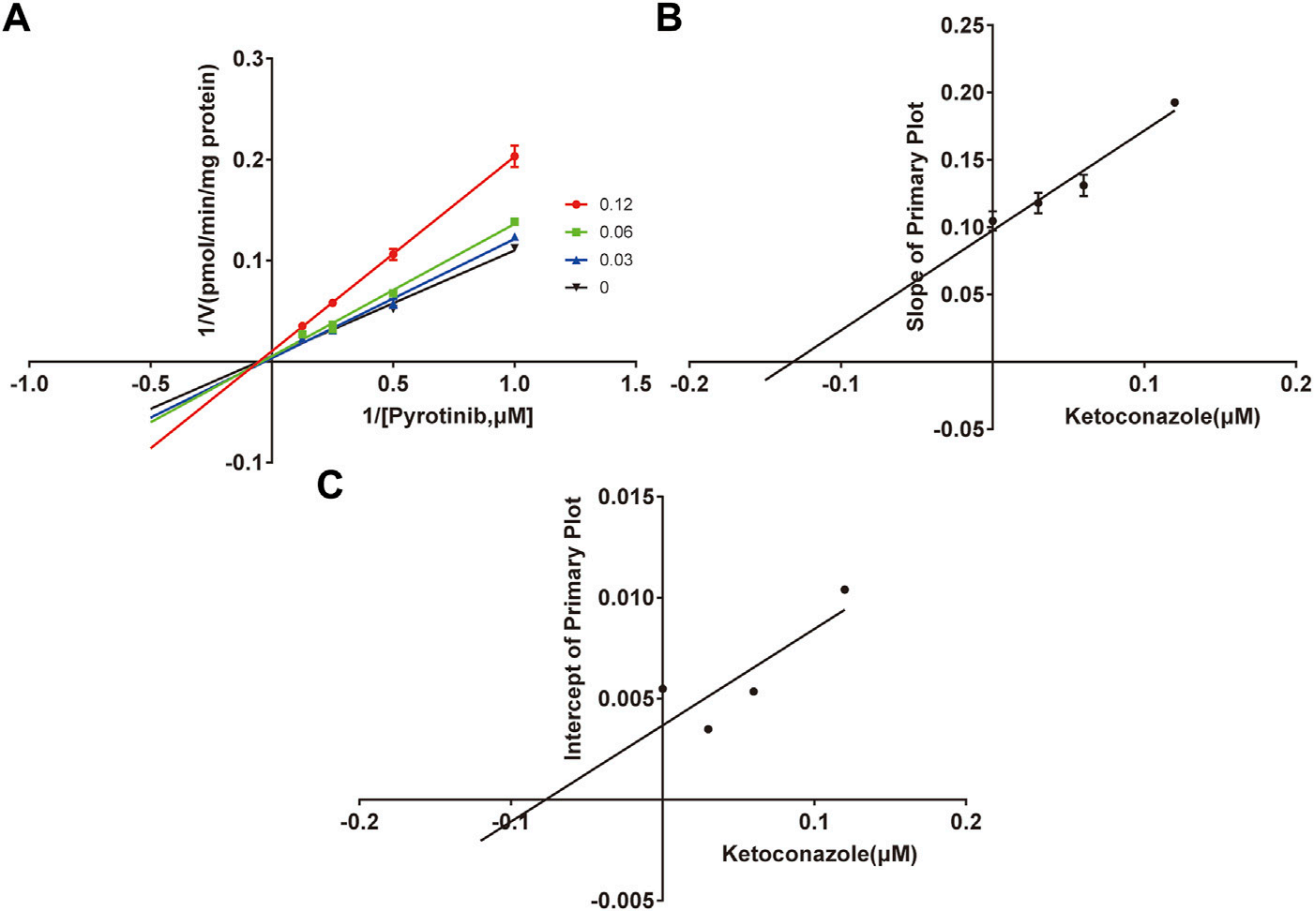
The Primary Lineweaver-Burk plots of ketoconazole inhibition on pyrotinib **(A)**; Slope of the primary plot of ketoconazole **(B)**; Intercept of the primary plot of ketoconazole **(C)** (values are means ± standard deviations, *n*=3).

**FIGURE 5:**
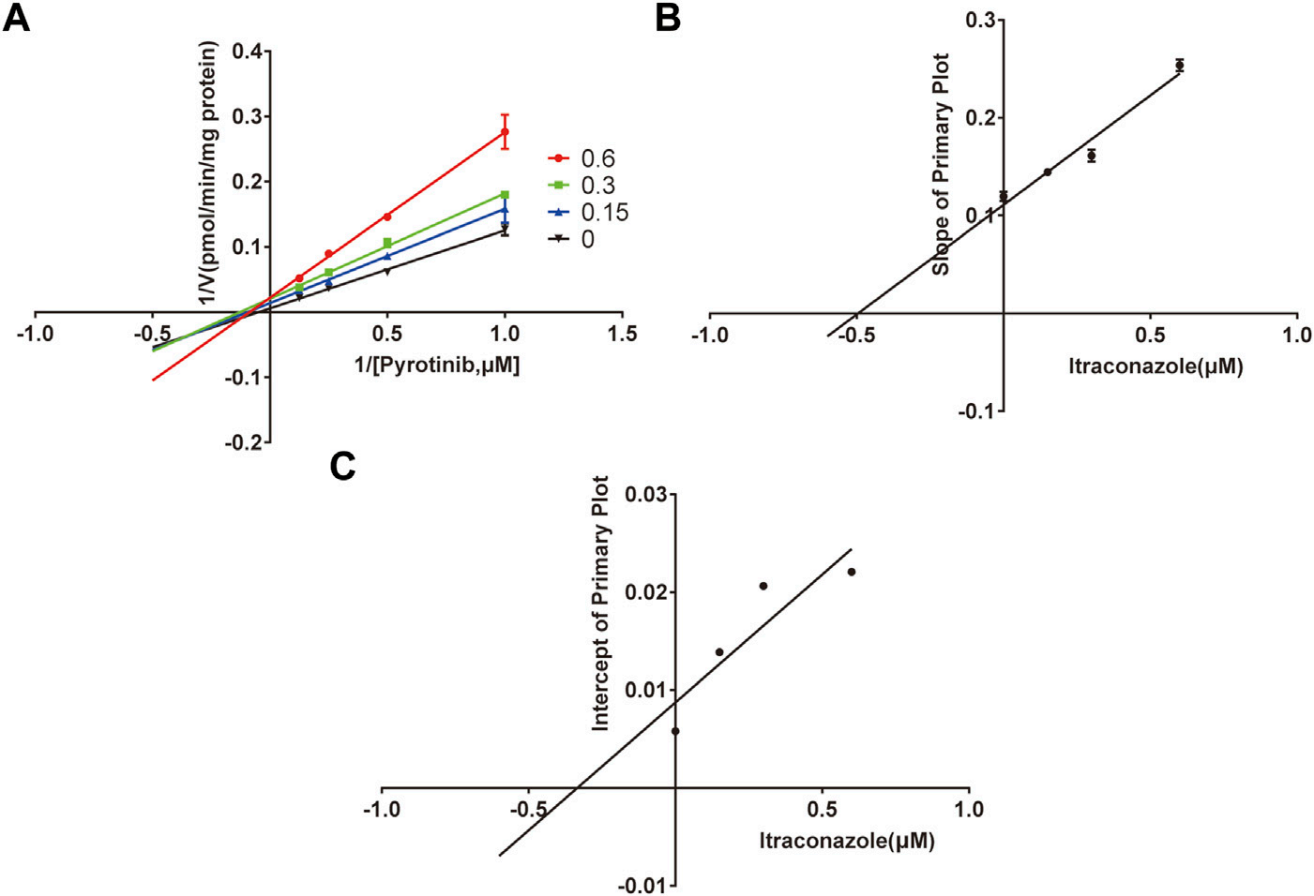
The Primary Lineweaver-Burk plots of itraconazole inhibition on pyrotinib **(A)**; Slope of the primary plot of itraconazole **(B)**; Intercept of the primary plot of itraconazole **(C)** (values are means ± standard deviations, *n*=3).

**FIGURE 6:**
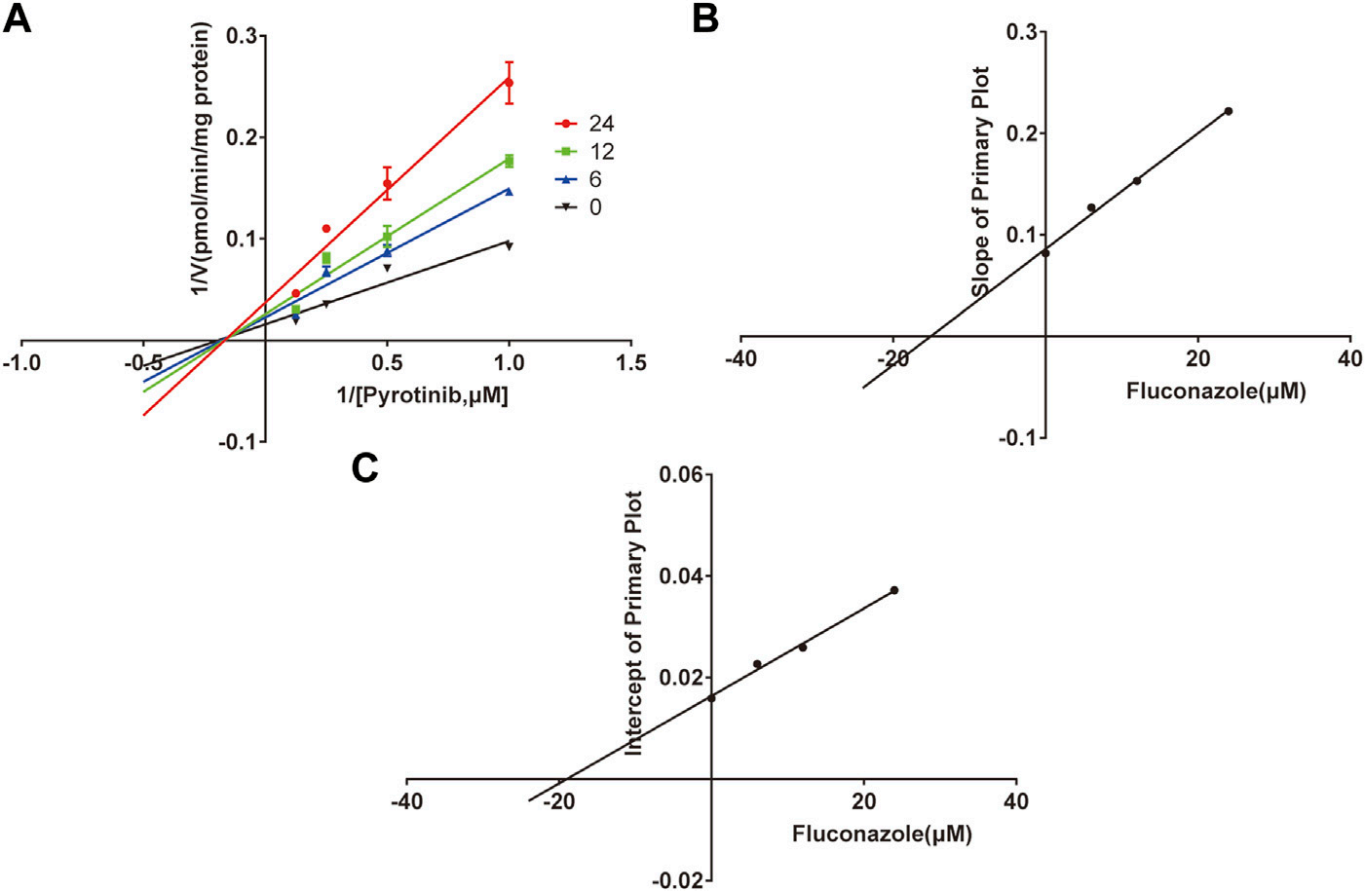
The Primary Lineweaver-Burk plots of fluconazole inhibition on pyrotinib **(A)**; Slope of the primary plot of fluconazole **(B)**; Intercept of the primary plot of fluconazole **(C)** (values are means ± standard deviations, *n*=3).

A quantification method for the detection of pyrotinib M1 and IS in RLMs was successfully established with no other interfering peaks, as shown in Figure 2. Pyrotinib’s K_m_ and V_max_ values were determined by nonlinear regression of the reaction velocity versus substrate concentration. As shown in Figure 3, V_max_ and K_m_ values of pyrotinib were 58.33 pmol/min/mg protein and 4.095 μM, respectively. Ketoconazole and itraconazole could strongly inhibit the metabolism of pyrotinib with IC_50_ value 0.06 and 0.27 μM, respectively. However, fluconazole only exhibited a relatively weak inhibition effect on pyrotinib (IC_50_ = 11.55 μM). As shown in Figures 4A, 5A, the Lineweaver-Burk plot showed that the family of straight lines intersected in the fourth quadrant, which indicated ketoconazole and itraconazole expressed mixed-type inhibition of noncompetitive/competitive. In detail, Ki and αKi (α = 0.583) values for ketoconazole were 0.132 and 0.077 μM, respectively. The Ki and αKi (α = 0.673) values for itraconazole were 0.496 and 0.334 μM, respectively. Fluconazole weakly inhibited pyrotinib with Ki and αKi (α = 1.259) values of 15.10 and 19.01 μM, respectively. The Lineweaver-Burk plot displayed that the family of straight lines intersected in the secondary quadrant in Figure 6A, indicating that fluconazole expressed mixed-type inhibition of noncompetitive/uncompetitive.

### 3.3 Effects of ketoconazole, fluconazole, and itraconazole on the metabolism of pyrotinib *in vivo*

**FIGURE 7:**
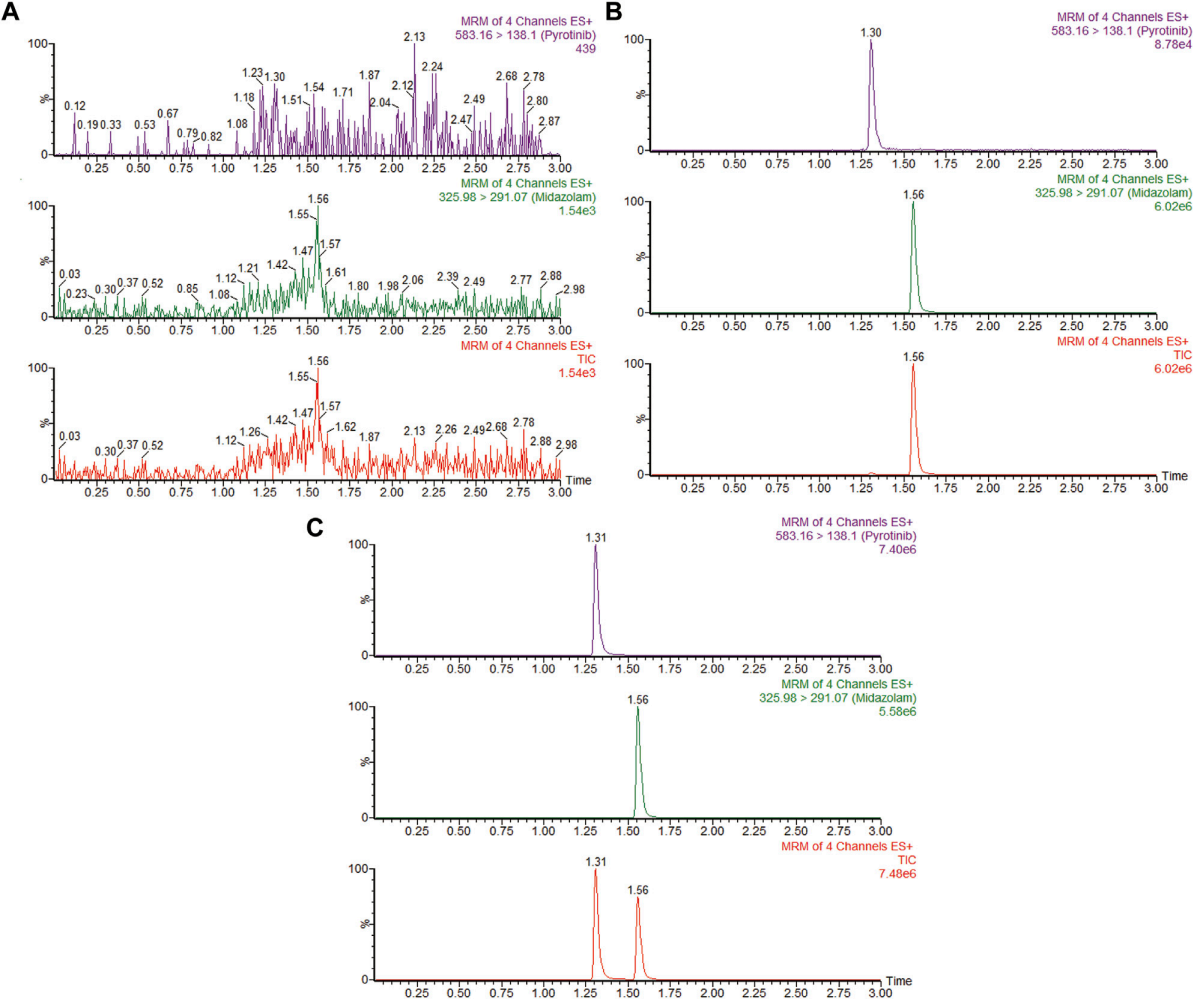
MRM chromatograms of pyrotinib and IS *in vivo*. **(A)** Blank rat plasma sample. **(B)** Denatured blank rat plasma sample with pyrotinib and IS. **(C)** Rat plasma sample after dosing pyrotinib with oral administration.

**FIGURE 8:**
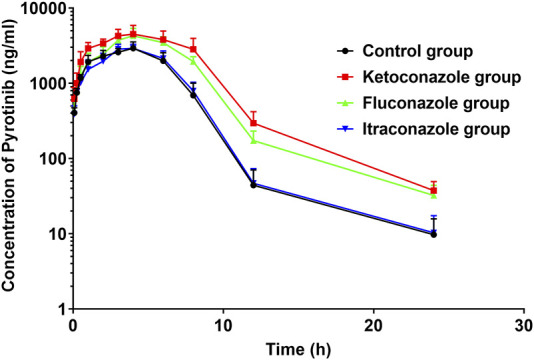
Average arterial plasma concentration-time curves of pyrotinib in rats in the Control group, ketoconazole group, fluconazole group and itraconazole group (*n* = 6).

The UPLC-MS/MS chromatogram results of pyrotinib and internal standard midazolam in blank rat plasma sample, denatured blank plasma samples pre-incorporated with pyrotinib and IS, and rat plasma samples after oral pyrotinib administration are shown in Figure 7. Blank plasma samples had a negligible effect on the peak shift during the elution time window, demonstrating no endogenous perturbation in the experimental samples. The pharmacokinetic parameter results revealed a significant difference between control group and ketoconazole or fluconazole treated groups (*p* < 0.05), especially for the values of AUC_(0-t)_, AUC_(0-∞)_, MRT_(0-t)_, MRT_(0-∞)_, t_1/2_, Cl/F, and C_max_ (Table 4). Mean plasma concentration-time curves of pyrotinib in rats of control group and experimental groups were shown in Figure 8. Compared with those of control group, the Cl/F value of pyrotinib in ketoconazole group decreased by 0.50-fold, but the AUC_(0-t)_ and C_max_ values were increased by 1.04-fold and 0.53-fold, respectively. Similarly, the Cl/F value of pyrotinib in fluconazole group decreased by 0.37-fold, but the AUC_(0-t)_ and C_max_ values were increased by 0.63-fold and 0.40-fold, respectively. These data suggest that ketoconazole and fluconazole could truly inhibit the metabolism of pyrotinib in rats. However, no significant difference could be found in pharmacokinetic parameters between the itraconazole and control groups, indicating that itraconazole had no inhibitory effect on pyrotinib *in vivo*.

**TABLE 4:** Main pharmacokinetic parameters of pyrotinib in the control group, ketoconazole group, fluconazole group and itraconazole group (*n* = 6, mean ± SD).

Parameter	Unit	Control	Ketoconazole	Fluconazole	Itraconazole
AUC_(0-t)_	ug/L*h	17,833.05 ± 2,953.65	36,304.55 ± 8,441.54*	29,069.82 ± 5,995.20*	18,002.86 ± 2,988.37
AUC_(0-∞)_	ug/L*h	17,840.03 ± 2,964.84	36,452.10 ± 8,474.10*	29,195.61 ± 6,033.37*	18,010.69 ± 2,979.02
MRT_(0-t)_	H	4.32 ± 0.40	5.38 ± 0.38*	5.28 ± 0.24*	4.55 ± 0.36
MRT_(0-∞)_	H	4.32 ± 0.42	5.47 ± 0.38*	5.37 ± 0.27*	4.56 ± 0.37
t1/2	H	1.26 ± 0.54	2.66 ± 0.20*	2.66 ± 0.15*	1.47 ± 0.63
T_max_	H	3.50 ± 1.23	4.00 ± 1.10	3.83 ± 0.41	4.00 ± 1.10
Vz/F	L/kg	0.81 ± 0.25	0.89 ± 0.24	1.09 ± 0.21	1.00 ± 0.58
Cl/F	L/h/kg	0.46 ± 0.08	0.23 ± 0.06*	0.29 ± 0.07*	0.45 ± 0.07
C_max_	ug/L	3,055.07 ± 358.30	4,680.18 ± 1,123.59*	4,291.18 ± 1,071.07*	3,004.65 ± 414.77

*p < 0.05, a significant difference compared to the control group (ANOVA).

## 4 Discussion

In this study, an UPLC-MS/MS based method was developed and validated to measure the concentration of pyrotinib and its metabolite, pyrotinib M1, simultaneously. Previous studies revealed that pyrotinib may get higher value of therapeutic efficacy when combined with other drugs. For example, pyrotinib combined with vinorelbine exhibited encouraging results in HER2-positive metastatic breast cancer, with little toxicity. Furthermore, this combination also showed promising antitumor activity in individuals with brain metastases (Li et al., 2021).

CYP3A4 is reported to be the main metabolic enzyme for pyrotinib; therefore, any CYP3A4 inducer or inhibitor may affect the metabolism of pyrotinib. Rifampicin, a strong index CYP3A4 inducer, has been shown to induce pyrotinib metabolism and decrease its exposure time in healthy adults (Cai et al., 2022). DDIs are very common among CYP3A4-mediated drugs but little attentions have been paid on the interactions between pyrotinib and azoles to date. In this study, effects of three azoles ketoconazole, fluconazole, and itraconazole on pyrotinib’s PK parameters were investigated both *in vitro* and *in vivo*.

Previous studies revealed that ketoconazole could increase the plasma exposure of ruxolitinib by 91% compared with ruxolitinib alone (Shi et al., 2011). Furthermore, ketoconazole could also interfere with the metabolism of one CYP3A4 probe drug midazolam (Lam et al., 2003). Similarly, our data indicated that ketoconazole could significantly inhibit pyrotinib metabolism both *in vitro* and *in vivo*. PK data in RLMs indicated that the inhibition effect of ketoconazole on pyrotinib belonged to the mixed inhibition type of noncompetitive/competitive, with an IC_50_ value of 0.06 μM IC_50_ < 1 mM indicates strong inhibitory efficiency based on the judgment. Similar to these data, ketoconazole have been reported to exhibit the stronger inhibition effect on CYP3A4 substrates than fluconazole or itraconazole, (Lou et al., 2019; Chen et al., 2020). Our *in vivo* experiment result also support this conclusion because when pyrotinib was coadministrated with ketoconazole in rat, its AUC_(0-t)_, AUC_(0-∞),_ and C_max_ values were significantly increased, while Cl/F value was decreased by approximately 50%, as compared to that of the control group. Therefore, ketoconazole’s negative effect on pyrotinib’s PK can be attributed to its inhibitory effect on CYP3A4 activity.

Fluconazole was reported to be a moderate CYP3A4/CYP2C9 inhibitor (Debruyne, 1997; Bellmann and Smuszkiewicz, 2017). In a previous study, coadministration of fluconazole with ruxolitinib could considerably increase systemic exposure to ruxolitinib and slowed down ruxolitinib’s elimination rate as compared with administrating ruxolitinib alone in healthy subjects (Aslanis et al., 2019). This study was similar to the results of our study, which the AUC_(0-t)_, AUC_(0-∞),_ and C_max_ values were significantly increased when coadministration of fluconazole with pyrotinib. Our *in vitro* data revealed that fluconazole could weakly inhibit the metabolism of pyrotinib with IC_50_ value of 11.55 μM, and the Ki and αKi (α = 1.259) values were 15.10 and 19.01 μM, respectively. This inhibition resulted from a mixed mechanism, incorporating uncompetitive and non-competitive inhibition. For the *in vitro* parameters, the apparent Ki values exhibited the dissociation constant for the interaction between the inhibitor and the enzyme, The parameter *α* was indicative of the inhibition type (Kim et al., 2019; Kim et al., 2021). When Ki = αKi, namely α = 1, it demonstrated noncompetitive inhibition. When α→0, it represented uncompetitive inhibition. When α→∞, it presented competitive inhibition. The mixed inhibition includes competitive, uncompetitive, and noncompetitive inhibition. When Ki > αKi, it showed the mixed inhibition of competitive and noncompetitive. When Ki < αKi, it represented mixed inhibition of uncompetitive and noncompetitive (Jin et al., 2015). The *α* value also represented the extent to which the binding affinity between enzyme and substrate is altered by the inhibitor. In our study, all three azole antifungals showed mixed inhibition. Ketoconazole and itraconazole revealed the mixed the inhibition of competitive and noncompetitive, with the value of *α* 0.583 and 0.673, respectively, while itraconazole represented the mixed inhibition of uncompetitive and noncompetitive.

Previous studies revealed that itraconazole was a potent CYP3A4 inhibitor (Prieto Garcia et al., 2018; Vishwanathan et al., 2018). In this study, itraconazole strongly inhibited pyrotinib *in vitro* with an IC_50_ value of 0.27 μM; however, it exhibited no effect *in vivo*. These results were similar to the previous study, where itraconazole had no effect or weak effect on the pharmacokinetic parameters of sunitinib and imatinib also mainly metabolized by CYP3A4 enzyme (Lin et al., 2014; Wang et al., 2021). Why the results of itraconazole *in vivo* were inconsistent with the results *in vitro*? It was reported that four isomers of itraconazole with different pharmacokinetic and pharmacological effects had been found, and different isomers of itraconazole have a significant distinction in the ability to inhibit the activities of CYP3A enzymes (Krasulova et al., 2019; Luo et al., 2020). This partly explains that although the itraconazole exerts strong CYP3A4 enzyme inhibitory activity *in vitro*, weaker inhibitory effect *in vivo* studies, which need further researches in the future.

## 5 Limitations

Current experiments are all based on animal models, and it could not fully simulate the actual state of DDIs in humans. Therefore, more work is still needed to further study the possible interaction mechanism between azole antifungal agents and pyrotinib in humans.

## 6 Conclusion

Ketoconazole showed more stronger inhibitory effects on pyrotinib metabolism than fluconazole, both *in vivo* and *in vitro*. Itraconazole had no effect on pyrotinib *in vivo* although it exhibited strong inhibitory effects *in vitro*. These data indicated that ketoconazole and fluconazole might be cautiously coadministered with pyrotinib in clinic, and a close therapeutic drug monitoring of pyrotinib concentration is suggested to avoid its potential adverse drug reactions.

## Data Availability

The original contributions presented in the study are included in the article/supplementary material, further inquiries can be directed to the corresponding authors.
